# Biomimetic Enamel Regeneration Using Self-Assembling Peptide P_11_-4

**DOI:** 10.3390/biomimetics8030290

**Published:** 2023-07-04

**Authors:** Mohammad Alkilzy, Ghalib Qadri, Christian H. Splieth, Ruth M. Santamaría

**Affiliations:** 1 Department of Preventive and Pediatric Dentistry, University of Greifswald, 17475 Greifswald, Germany; 2 Department of Orthodontic and Pediatric Dentistry, Arab American University, Jenin P.O. Box 240, Palestine

**Keywords:** biomimetic, enamel regeneration, P_11_-4, initial caries, self-assembling peptide

## Abstract

The recent understanding of the etiology and pathology of dental caries has shifted its treatment from invasive drill and fill conventional strategies to noninvasive and/or minimally invasive approaches. Guided tissue regeneration (GTR) is a well-established therapeutic approach in medicine and periodontal and oral surgery. Recently, the concept of biomimetic regeneration has been further expanded to treat the loss of hard dental tissues. Self-assembling peptides have emerged as a promising biomaterial for biomimetic regeneration due to their ability to construct a protein scaffold in the body of early carious lesions and provide a matrix that promotes remineralization. This review article accompanies the development of self-assembling peptide P_11_-4 for the treatment of initial carious lesions. In vitro and in vivo studies on the safety, clinical applicability, and efficacy of P_11_-4 are discussed. Furthermore, different treatment options and potential areas of application are presented.

## 1. Introduction

Dental caries is still the most prevalent disease in the world, with a recent global prevalence of ~35% of people having untreated cavities, even though it has declined in many countries [1,2,3]. Dental caries is a pathophysiological process in the dental biofilm that causes an imbalance in the demineralization–remineralization equilibrium, resulting in a net loss of tooth minerals, and ultimately leading to the formation of cavities [4]. Hence, the traditional treatment of carious lesions by merely drilling and filling constitutes a repair of the damage; however, it does not address the underlying disease itself. Furthermore, invasive restorative interventions might lead to the damage of healthy tooth-surrounding tissue, which destabilizes the natural tooth structure and creates a vicious cycle of new restorations, thus resulting in the removal of more dental tissue when replacement of existing restorations is required. In this context, the most appropriate strategy for caries control should involve shifting the balance toward remineralization by improving daily oral hygiene and applying remineralization agents, such as fluoride. 

On the other hand, as caries lesions progress gradually through stages over the time, preventive treatment strategies in the early stages of caries lesions are more realistic than and preferable to restorative treatments [5]. Several noninvasive and minimally invasive treatments have been suggested for early caries stages, prior to cavitation of the enamel surface, to avoid or delay restorations [6]. Examples of these treatments include sealing carious lesions (fissure sealants and resin infiltration), remineralization procedures mainly using fluorides (varnishes, gels, and toothpaste) [7,8], CPP/ACP (casein phosphopeptide/amorphous calcium phosphate) vehicles alone or in combination with fluoride interventions [9], and hydroxyapatite toothpaste, which has shown positive effects in enamel remineralization and the prevention of dental caries [10]. Additionally, diet control and regular biofilm removal enable natural remineralization of carious lesions through saliva [11].

The main objective of this review is to provide a foundation of knowledge on self-assembling peptide P_11_-4, along with its various aspects used for enamel regeneration, and to examine how they can be categorized as biomimetic approaches. The review entails a thorough and comprehensive analysis of both past and recent studies that have investigated this topic. Moreover, it aims to shed light on the specific approaches utilized, the materials employed, and the clinical applications associated with enamel regeneration. By examining these aspects, a deeper understanding of the biomimetic nature of enamel regeneration can be achieved.

## 2. Regeneration of Dental Tissues

Regenerative medicine is an innovative approach that enables the replacement of damaged or diseased tissues by leveraging the biological healing process and supporting this process beyond its natural capacity. In recent years, there has been a shift from traditional reparative dentistry to regenerative dentistry, which reflects the current trend in medicine [12]. Unlike bone, the rich cellular and vascular tissue where regenerative approaches have been well established, dental hard tissues, especially enamel, have limited regenerative capabilities as they lack any regenerative cells or vascularity. On the other hand, the dental hard tissue remineralization process refers to the natural process of restoring minerals to the tooth structure. It is a vital process in maintaining the health and integrity of dental tissues, particularly enamel. Both terms, “regeneration” and “remineralization”, are often used interchangeably in the literature of regenerative dentistry. However, the term “regeneration” refers to the broader process of tissue replacement and repair, encompassing the restoration of both structure and function. On the other hand, the term “remineralization” specifically relates to the restoration of minerals to dental tissues.

The natural remineralization process of enamel is solely driven by saliva and is, thus, uniquely different from all other tissues. In addition, the enamel matrix, which guides mineralization during odontogenesis, is degraded during tooth maturation prior to eruption and is, therefore, not available to guide remineralization. To address this challenge, a new treatment modality called biomimetic mineralization has emerged. This innovative approach mimics the function of the enamel matrix during the natural mineralization process and supports the regeneration process driven by saliva beyond its natural capacity [13]. Oligomeric β-sheet-forming peptides were introduced as a biomimetic mineralization approach for the treatment and prevention of dental caries. These short-chain 11-amino-acid peptides possess a unique ability to self-assemble into three dimensional fibrillar scaffolds [14].

Studies have shown that treatment with these peptides significantly increases net mineral gain in dental tissues [13,15]. This effect is achieved through a combined effect of increased mineral gain and inhibition of mineral loss. Moreover, the self-assembling peptide has been demonstrated to induce de novo hydroxyapatite formation, thus forming new mineral structures that are chemically and structurally very similar to natural enamel [15,16].

In tissue engineering, the need for custom-made biomimetic scaffolds has risen to gain faster and better repair results. Therefore, the scaffold design should be optimized to meet the desired biological requirements [17]. In the secluded space within an initial carious lesion, a biomimetic scaffold can be used to facilitate natural hard tissue remineralization through saliva, resulting in guided enamel regeneration, analogous to guided tissue regeneration and guided bone regeneration [18]. Biomimetic remineralization follows the concept of mimicking the natural process of remineralization in the treatment of dental caries. It seeks to remineralize initial carious lesions, rather than sealing or infiltrating them with substitute materials.

## 3. Self-Assembling Peptide P_11_-4 for Enamel Regeneration

The self-assembling peptide P_11_-4 is a rationally designed peptide. Its chemical structure contains 11 amino acids (Ace–Gln–Gln–Arg–Phe–Glu–Trp–Glu–Phe–Glu–Gln–Gln–NH_2_), and it has the ability to self-assemble into higher-order structures such as tapes, ribbons, fibrils, and fibers. These higher-order structures can mimic the enamel matrix structure of tooth enamel [13,19,20].

If the P_11_-4 complex, formed through the self-assembly of the oligomeric β-sheet-forming peptides, exhibits a similar acicular shape, nanometric size, and crystallinity to natural enamel, it can be considered biomimetic. This biomimetic nature allows the P_11_-4 complex to closely resemble the mineral structure of enamel and potentially support the regeneration and remineralization of dental hard tissues [21].

P_11_-4 was also designed and screened for properties that make it suitable for remineralization, such as its high affinity to hydroxyapatite and its potential to nucleate hydroxyapatite [22].

Assembled P_11_-4 forms scaffold-like structures with negative charge domains, mirroring biological macromolecules in mineralized tissue extracellular matrices (ECMs) [15]. In a biomimetic strategy based on recapitulating normal enamel histogenesis, following P_11_-4 self-assembly, the anionic groups of the P_11_-4 side-chains would attract Ca^++^ ions, inducing de novo precipitation of hydroxyapatite [13]. The application of fluoride provides further fluoride ions for the remineralization process.

**Figure 1:**
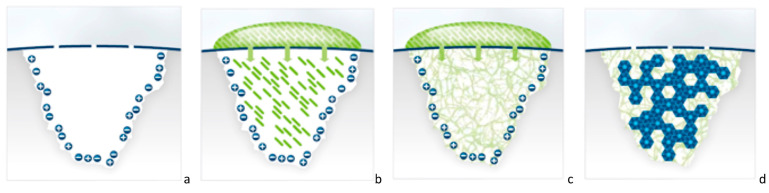
(**a**–**d**) Cartoon illustration explaining the mechanism of action of P_11_-4 in the treatment of initial caries regenerating the enamel by the self-assembling peptide P_11_-4. (**a**) Initial carious lesion, where the broken blue line represents the mineralized surface layer and pores that connect the lesion’s body with the oral environment. The subsurface carious lesion is charged negatively and positively with free ions. (**b**) A drop of monomeric self-assembling peptide P_11_-4 solution is applied on the lesion surface, and the monomers diffuse into the lesion. (**c**) Due to the higher ionic strength and the acidic pH of the lesion, P_11_-4 assembles and builds a 3D scaffold within the lesion. (**d**) De novo hydroxyapatite crystals (in blue) form around the self-assembling peptide scaffold.

The mechanism of action is illustrated in Figure 1a–d. The safety and efficacy of P_11_-4 in enhancing enamel remineralization have been evaluated in a series of in vitro and in vivo studies, including multiple randomized clinical controlled trials.

**Figure 2:**
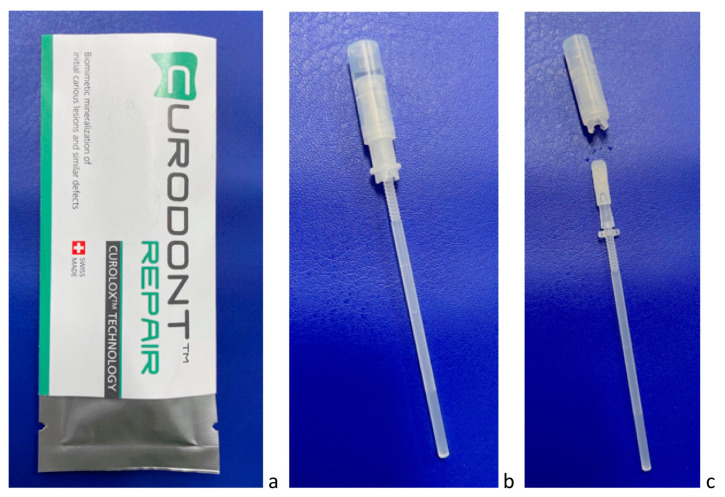
(**a**–**c**) P_11_-4 is provided for dentists as Curodont™ Repair (**a**), which is administered as water solution using an applicator (**b**,**c**).

Numerous studies have been conducted on the self-assembling peptide P_11_-4, both in vitro and in vivo, to investigate its potential for biomimetic remineralization and enamel regeneration. These studies have demonstrated that one of the major advantages of self-assembling peptides is their ability to form a biomimetic scaffold within subsurface caries lesions. This scaffold relies on the natural mineralization processes in saliva to promote remineralization and regeneration of the lesion [13,14,19,20,22]. This approach presents a novel possibility for dentists to treat carious lesions in their early stages using a minimal invasive and biologically focused treatment [23]. Self-assembling peptide P_11_-4 is currently commercially available as Curodont™ Repair (Credentis AG, Windisch, Switzerland, Figure 2).

## 4. Safety, Biocompatibility, and Clinical Feasibility of P_11_-4

Evaluating the biocompatibility of biomaterials is essential to ensure their safe application within the human body from a biomedical perspective [24]. Biocompatibility refers to the ability of a material to perform its desired function without eliciting adverse reactions or harm to the surrounding tissues or the overall physiological system. When considering the use of self-assembling peptide P_11_-4 for enamel regeneration or any other biomedical application, it is important to assess its safety profile and compatibility with human tissues.

Investigating the biocompatibility, safety, and clinical applicability of new dental materials is required in order to gain market approval. According to ISO 10993 or equivalent, biocompatibility studies showed that P_11_-4 did not raise any cytotoxic effects or any immunologic response [19,20,25]. Potential risks related to the application of P_11_-4 are considered to be minor as it is applied onto an a cellular enamel tissue; thus, any physiological interaction at the treatment site is unlikely. Even if swallowed, P_11_-4 will be degraded into natural amino acids or secreted. Furthermore, the amount used per patient is extremely small.

P_11_-4 has been also proven safe in patients in in vivo studies, with no adverse events, medical complications, or allergic reactions related to treatments (during or post treatment up to 12 months follow-up) [14,23,26]. In addition, there is evidence from some of the clinical studies where the majority of patients reported comfort and high satisfaction following the treatment with P_11_-4 product (Curodont™ Repair), with all patients being willing to repeat such minimally invasive treatment. Likewise, treating dentists reported that the application of Curodont™ Repair was easier than placing a filling or a fissure sealant [23].

## 5. In Vitro Studies on Remineralization Effect of P_11_-4

The diffusion of Peptide _11_-4 into carious lesions has been extensively studied in vitro, with results indicating that it diffuses beyond the volume of the lesion into the enamel layer below [22]. Kind et al. [22] used time-resolved confocal microscopy to investigate the diffusion into the caries lesion and found that about 35% of P_11_-4 remained within the artificial caries lesions, forming a scaffold that enabled de novo hydroxyapatite crystal formation. P_11_-4 assembled into fibers presents clusters of negative charges made up of four Glu-residues on its surface, presenting a potential Ca^2+^-binding site. This affinity of P_11_-4 for Ca^2+^ ions and its ability to act as a nucleation site for de novo hydroxyapatite crystals has been confirmed by several in vitro studies [13,22,27].

In a study conducted by Kirkham et al. [13], electron micrographs were obtained of undiluted P_11_-4 gels used in de novo precipitation experiments. After the experiments, ceratin gels were fixed and stained. The results revealed the presence of fibrils within the gel, arranged in twisted bundles consisting of 20–100 aligned fibrils. These bundles exhibited widths ranging from hundreds of nanometers to micrometers, with lengths extending to several micrometers. The structure of these bundles bore a resemblance to collagen fibers. Following in vitro mineralization, electron-dense deposits were observed in an association with the bundles, potentially indicating sites where hydroxyapatite nucleation had occurred [13]. Additionally, two studies [28,29] employed microtomography to analyze the potential for remineralization, and their findings demonstrated resulting mineral density levels reaching up to 90% of the original enamel density.

In another study conducted by Sindhura et al. [16], the remineralizing efficacy of self-assembling peptide (SAP) P_11_-4 was qualitatively and quantitatively evaluated using scanning electron microscopy (SEM) and energy-dispersive X-ray (EDX). Enamel samples were allocated into two groups: the test group, treated with SAP P_11_-4, and the control group, treated with casein phosphopeptide/amorphous calcium phosphate (CPP/ACP). The samples were then stored in artificial saliva to assess the remineralizing efficacy at 1 week, 1 month, and 3 month intervals. Samples treated with CPP/ACP exhibited a significant increase in Ca:P ratio (2.04 ± 0.2) with irregular surface calcific deposition at the 1 week interval. However, this deposition reduced the Ca:P ratio over time (1.87 ± 0.11 observed at the 3 month interval. On the other hand, P_11_-4 treatment showed a significant increase in the Ca:P ratio (1.95 ± 0.10) with uniform ion deposition, indicating hydroxyapatite nucleation, over the course of 3 months. The authors concluded that SAP P_11_-4 demonstrated superior remineralization with uniform mineral deposition compared to CPP/ACP at the 3 month interval [16].

## 6. In Vivo and Clinical Studies on Therapeutic Effect of P_11_-4

After confirming the safety and remineralization effect of P_11_-4 on dental carious lesions in vitro [13], the first in-man clinical trial started with the treatment of 15 healthy adults [14]. This noncontrolled clinical trial included patients with buccal white spot lesions. They received a single treatment of P_11_-4, and changes in the lesion were observed clinically for 6 months. Most of the lesions were inactive, yet the study could show a significant decrease in lesion size as judged by VAS (*p* = 0.02) and shifted the progression of lesions to remineralizing (*p* < 0.001) [14].

Shortly after the first in-man trial, a second uncontrolled trial was performed on proximal lesions of 26 patients with 35 carious lesions. The lesions were clinically followed for 1 year with clinical bite-wing X-rays [30]. As assessed on both clinical radiographs and digital subtraction radiographs in a combined score, a single dose of P_11_-4 resulted in regression of 20/28 lesions, no change in 4/28 lesions, and progression in 4/28 lesions, of which one lesion progressed significantly. The behavior of the lesions was independent of their initial lesion stage, i.e., whether the lesion had reached into the dentin or not. The analyses were conducted via blinded assessors as automatic X-ray analyses were not available at the time. This study was the only clinical study conducted with P_11_-4 on proximal caries and followed up with X-rays.

After those initial uncontrolled safety trials, a number of randomized clinical studies were conducted in order to investigate its clinical effectiveness and efficacy in dental patients [23,26,31].

In the first randomized controlled trial, Alkilzy et al. [32] investigated the safety and clinical efficacy of P_11_-4 for the treatment of initial occlusal caries in children with visible active early lesions on erupting permanent molars. Patients were randomized to either the test group (P_11_-4 + fluoride varnish) or control group (fluoride varnish alone). Caries lesions were assessed at baseline and at 3- and 6 months post treatment using laser fluorescence, a visual analog scale, the International Caries Detection and Assessment System (ICDAS), and the Nyvad caries activity criteria. In comparison with the control group, the test group showed clinically and statistically significant improvement in all outcomes at the 3 and 6 month follow-ups. The laser fluorescence readings (odds ratio = 3.5, *p* = 0.015) and visual analog scale scores (odds ratio = 7.9, *p* < 0.0001) were significantly lower for the test group, and they showed regression in the ICDAS caries index (odds ratio = 5.1, *p* = 0.018) and conversion from active to inactive lesions according to the Nyvad criteria (odds ratio = 12.2, *p* < 0.0001). Furthermore, no adverse events were reported. The authors concluded that the biomimetic mineralization by P_11_-4 in combination with fluoride application presents a simple, safe, and effective noninvasive treatment for early carious lesions, which means avoidance of the additional loss of dental hard tissue in the case of restorative treatments, thus prolonging teeth lifespan and lowering dental treatment costs.

In 2020, a similar clinical trial was published, which can be viewed as an extension to this first clinical trial by Alkilzy et al. [23]. Doberdoli et al., 2020 [31] had the same control group (fluoride varnish) and test group 1 (P_11_-4 and fluoride varnish), but extended the design with a second test group (P_11_-4 monomeric and preventive Gel with P_11_-4 polymeric). The study design was a prospective randomized controlled three-arm clinical trial with patient blinding. The results were comparable with those of Alkilzy et al. [23], yet inactivation of P_11_-4-treated teeth according to the Nyvad Caries Activity Criteria was even more prominent, with 56/56 lesions seen as inactive compared to 13/23 for the control group. The laser fluorescence value increased for the control group within the 1 year follow-up period (Day 0. 27.2 ± 6.6; Day 360, 31.8 ± 6.4). Interestingly the two test groups did not differ significantly from each other (test group 1: Day 0, 31.4 ± 6.5; Day 360, 22.7 ± 4.3 and test group 2: Day 0, 25.3 ± 7.1; Day 360, 17.6 ± 5.1), yet both demonstrated a superior reduction in laser fluorescence compared to the control group (*p* < 0.0005). Both control groups showed significantly higher regression in ICDAS scores (*p* < 0.05). In the control group, 16/23 lesions remained unchanged and 7/23 increased in ICDAS score, whereas none regressed. In test group 1, 2/27 lesions showed regression and 25/27 remained unchanged. In test group 2, 6/27 regressed and 21/27 remained unchanged. No lesion in the test groups progressed into a larger ICDAS class. The authors concluded that guided enamel regeneration with P_11_-4 was successful, and that fluoride varnish could be replaced by a twice-weekly application of a P_11_-4-containing gel.

A prospective, randomized, split-mouth clinical trial was conducted to compare the efficacy of P_11_-4 and fluoride varnish in treating early buccal caries lesions [26]. Standardized photographs were taken at baseline and during follow-up visits up to 1 year. The decrease in lesion size between the test and the control groups was assessed morphometrically by a blinded assessor. The results revealed a significant difference between the test and control groups, indicating a reduction in lesion size for the test lesions and stabilization for the control lesions. Interestingly, some of the treated lesions already showed micro-cavities at baseline. These micro-cavities exhibited minimal growth in the test group but significantly extended in the control group. Since only a few cases exhibited this phenomenon in both groups, no statistical analyses were performed. However, further investigation in this area would be of interest. The authors concluded that the size of early carious lesions treated with P_11_-4 was significantly reduced, and this reduction was superior to that achieved with fluoride varnish treatment. Additionally, a second application of P_11_-4 after 90 days was explored in this clinical trial. However, on the basis of the regression curve during the one-year follow-up, it is not evident that this second application provided a notable benefit.

Another clinical trial was conducted by the Sedlakova et al. [33]. This trial had a more complex design as a randomized, placebo-controlled, blinded split-mouth clinical trial with a sequential design. The trial had a total follow-up of 9 months. The test group received P_11_-4 treatment at baseline whereas the control group received a sham treatment including a placebo. Both treatment groups received fluoride varnish on day 90. The design enabled the testing of four hypotheses according to the progression and regression of the white spot lesions as measured morphometrically on standardized photographs by a blinded assessor with all data given as normalized data. P_11_-4 showed, over a 90 day period, a significantly higher decrease in lesion size compared to placebo (−0.19 ± 0.25 vs. −0.07 ± 0.24; *p* = 0.008) and a significantly higher decrease in lesion size compared to fluoride varnish (−0.19 ± 0.25 vs. −0.03 ± 0.13; *p* < 0.001). Furthermore, P_11_-4 and fluoride varnish showed, over a 180 day period, a significantly higher decrease in lesion size compared to fluoride varnish alone (−0.20 ± 0.28 vs. −0.04 ± 0.19; *p* = 0.003). Lastly, due to the complex study design, the data could demonstrate that the application of fluoride varnish was not impeded or accelerated by the previous addition of P_11_-4. Fluoride varnish with prior application of P_11_-4 (−0.06 ± 0.18) showed no difference to fluoride varnish alone (−0.04 ± 0.19). The first three comparisons corroborate the findings of Alkilzy et al. [23], Doberdoli et al. [31], and Bröseler et al. [26], whereas the last finding is essential for clinical application, as the use of fluoride varnish is widely used in dentistry, and a possible detrimental effect of the combination with P_11_-4 would have prohibited use of P_11_-4 in the clinic. The authors concluded that P_11_-4 supports the formation of de novo hydroxyapatite crystals deep within and throughout the carious lesion body, thus offering the clinician a new, effective, non-aerosol-generating, and noninvasive treatment option [33].

A study published in 2020 by Welk et al. [34] investigated P_11_-4 treatment of fresh orthodontic lesions. The study included 23 patients that showed buccal white spot lesions after debonding with a follow-up of 180 days. The study design was a split-mouth conventional treatment-controlled clinical trial. The primary parameter was the impedance measurement of the white spot lesion (CariScan), and the secondary parameter was the morphometric assessment of the lesion size (measured by the shadepilot). Both primary and secondary parameters showed superior regression of the test group lesions treated with P_11_-4 compared to the control lesions receiving standard care. Impedance measurements were lower in the test group compared to control group values (after 180 days): −14.6 (95% CI: −24.5, −4.8) (*p* = 0.007). Morphometric measurements differed in the test group compared to control group values (after 180 days): −1.0 (95% CI: −1.6, −0.4) (*p* = 0.004). The authors concluded that the treatment of initial carious lesions with self-assembling peptide P_11_-4 led to superior remineralization of the subsurface lesions compared with the control teeth [34].

In another clinical trial, Gohar et al. [35] investigated P_11_-4 treatment compared to fluoride varnish in the progression/regression of post-orthodontic white spot lesions in 58 patients (29 per group). In this randomized controlled clinical trial, the effect was investigated by laser fluorescence and ICDAS, and then followed up after 3 (T1) and 6 (T2) months. The laser fluorescence values showed a significantly higher decrease for the P_11_-4 group than for the control group with fluoride varnish (P_11_-4: (T0) 14.29 ± 3.97, (T2) 6.45 ± 1.99; fluoride varnish: (T0) 16.46 ± 2.91, (T2) 10.51 ± 1.94; *p* < 0.001). The changes in ICDAS values did not show significant differences between the groups. This might be due to the fact that only ICDAS 1 and ICDAS 2 lesions were included in the study, whereas, in the other clinical studies using ICDAS as parameters (Alkilzy et al. [23] and Doberdoli et al. [31]), more ICDAS 3 cases were included, which showed a higher tendency to regress to a lower class than ICDAS 2 cases. The authors concluded that the biomimetic remineralization promoted by P_11_-4 achieved successful subsurface remineralization, making the material a promising guide to lesion regression in post-orthodontic therapy [35].

A similar study in the treatment of post-orthodontic lesions was performed by Kobeissi et al. [36]. The study design was a split-mouth controlled trial that included nine patients and 40 young permanent teeth with early white spot lesions, followed up for 6 months. As in Gohar et al. [35], fluoride varnish was used as treatment for the control group. The lesion progression/regression was followed up with laser fluorescence and ICDAS assessments. Laser fluorescence values showed significantly higher reduction for P_11_-4-treated lesions than fluoride varnish-treated lesions (P_11_-4: (baseline) 10.35, (6 months) 5.85; fluoride varnish: (baseline) 9.45, (6 months) 6.40; *p* = 0.0124). The progression of ICDAS classification of the white spot lesions decreased for both test and control groups as can be expected for white spot lesions. The regression in ICDAS classifications between the two groups did not vary significantly. The authors concluded that P_11_-4 showed superiority due to its guided enamel regeneration potential, which can open the doors to a new concept in enamel healing [36].

In contrast to the clinical trials mentioned above are the results of the clinical trial by Gözetici et al. [37]. This study on 21 patients was performed as a four-arm split-mouth randomized clinical trial comparing resin infiltration, P_11_-4, fluoride varnish, and a no-treatment control. The lesion progression/regression was followed by laser fluorescence and LAA-ICDAS. The laser fluorescence values for P_11_-4 reduced from baseline to 6 months from 24.4 ± 13.55 to 16.25 ± 14.00, which is comparable to data reported in other studies. However, in the study by Gözetici et al. [37] the fluoride varnish group reduction was more pronounced from 21.5 ± 11.38 to 11.4 ± 9.18, which is in contrast to the results from the studies described above. The no-treatment control only exhibited a slight reduction in laser fluorescence from 17.95 ± 6.26 to 13.8 ± 10.65. The most pronounced reduction was seen in the resin infiltration group from 33.2 ± 17.33 to 9.95 ± 9.11, which is not surprising as resin infiltration will kill the bacteria within the lesion and the natural decay of porphyrins which cause the laser fluorescence dominates this value. Of more concern is the highly significant difference in the baseline value among the groups (*p* = 0.004), which was not statistically accounted for. Thus, comparisons among the groups could not be performed.

There are numerous clinical trials reported in the literature that have utilized P_11_-4. However, this review did not discuss some of these trials in details due to incomplete data, small patient samples, or indications that were not within the scope of this review.

For example, Singh et al. [38] investigated the use of P_11_-4 on teeth affected by molar incisor hypomineralization (MIH), which is not a caries indication. Therefore, it did not fully fit the scope of this review. Kamh et al. [39] conducted a three-arm study that investigated P_11_-4 (Curodont™ Repair), sodium fluoride agent (Lunos^®^ Polierpaste), fluoride, hydroxyapatite, and xylitol paste (Remin Pro forte), and measured the changes via ICDAS scores. All three groups showed a pronounced reduction in ICDAS scores [39].

Aziz et al. [40] conducted an uncontrolled trial that investigated P_11_-4 in post-orthodontic lesions. This study, as other uncontrolled trials, should be interpreted with caution, requiring confirmation through further investigation in larger, well-designed studies.

Lastly, Riad et al. [41] reported on a split-mouth study that compared P_11_-4 (Curodont™ Repair) to a single-dose fluoride varnish (Duraphat™) and assessed the changes using a visual analog scale. The authors concluded that P_11_-4 offers a therapeutic option for enamel regeneration, by providing a scaffold for improved remineralization of the lesion body, forming new enamel, which enhances the masking of white spot lesions.

Table 1 presents the pioneer studies investigating the effect of P_11_-4 in the biomimetic enamel regeneration and summaries its main conclusions (Table 1).

## 7. Perspectives for Treatments with P_11_-4

The previously mentioned studies demonstrated the ability of P_11_-4 to enable biomimetic regenerative remineralization of initial caries lesions. Furthermore, a study investigated an additional treatment approach with P_11_-4 to treat dentin hypersensitivity [45].

Another treatment approach of P_11_-4 might be tooth whitening. A feasibility in vitro study on tooth whitening reported a positive whitening effect of P_11_-4 [46].

An in situ study by Jablonski-Momeni et al. [43,47] investigated the remineralizing effect of P_11_-4 compared to fluoride applied to tooth surfaces with orthodontic brackets. The results showed significantly higher remineralization in the P_11_-4 group than in fluoride group (*p* = 0.003) [43]. These results are promising for use of the self-assembling peptide P_11_-4 as a preventive or therapeutic measure in accompanying orthodontic treatments to prevent the formation of white spots around orthodontic brackets or to treat and mask them. A randomized clinical trial supported this therapeutic effect of P_11_-4 on white spot lesions after orthodontic treatment with brackets. The impedance and the morphometric measurements to determine the size of white spot lesions showed that the treatment of initial carious lesions with self-assembling peptide P_11_-4 led to superior remineralization of the subsurface lesions compared with the control teeth [34].

In addition to the above findings, further investigations were conducted to explore the interaction of P_11_-4 with dentin, as well as its impact on the proteolytic activity, mechanical properties of the bonding interface, and nano-leakage evaluation in artificial caries-affected dentin. The results showed that P_11_-4 interacts with collagen type I, effectively enhancing the resistance of collagen fibers to proteolysis. Moreover, it was observed that P_11_-4 improves the stability of the hybrid layer formed in artificial caries-affected dentin [48]. Another study highlighted that the incorporation of self-assembling peptide P_11_-4 into the bonding agent significantly increased bond strength to demineralized dentin (*p* < 0.05). This suggests that, by modifying the dentine surface and restoring conditions resembling sound dentin, the interfacial bonding can be enhanced [27]. Overall, these findings indicate that P_11_-4 holds promise in terms of its ability to interact with dentin, strengthen collagen fibers, and improve bonding properties at the interface between restorative materials and caries-affected dentin [27].

Table 2 shows some indications and limitations for minimally invasive caries treatment using self-assembling peptide P_11_-4.

## 8. Clinical Application of P_11_-4 on Carious Lesions

**Figure 3:**
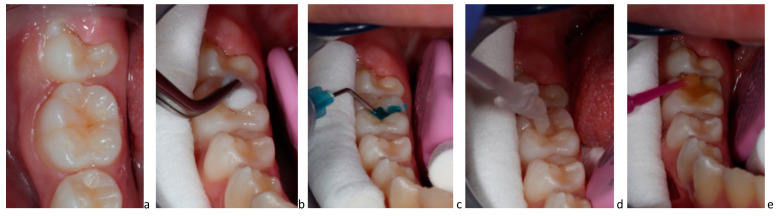
(**a**–**e**) Clinical application of P_11_-4: (**a**) initial active carious lesion in a permanent molar; (**b**) cleaning of organic debris with 3% sodium hypochlorite; (**c**) etching with 35% phosphoric acid; (**d**) application of P_11_-4 and waiting for 3–5 min for diffusion into the lesion; (**e**) fluoride varnish application.

P_11_-4 has been successfully introduced for clinical use by dentists (Curodont™ Repair) [36,42,44]. The application technique, according to the manufacturer’s instructions, is illustrated step-by-step for treatment of an early carious lesion on a first permanent molar during the eruption period (Figure 3) [23,32,49].

As the Curodont™ Repair has to be applied as water solution, its application is more tolerant to moisture than adhesive sealing or infiltration using resin-based materials.

## 9. Conclusions

The self-assembling peptide P_11_-4 has recently emerged in dentistry as a very promising biomaterial for biomimetic regeneration due to its potential to mimic the enamel extracellular matrix. Overall, P_11_-4 shows favorable outcomes for minimally noninvasive treatment of initial carious lesions. Nevertheless, further research is needed to determine its long-term efficacy in various conditions.

## Figures and Tables

**Table 1:** Pioneer studies investigating the effect of P_11_-4 in biomimetic enamel regeneration.

Author, Year	Type of the Study	Main Conclusions
Kirkham et al., 2007 [13]	In vitro	P_11_-4 enhances remineralization and induces de novo hydroxyapatite nucleation
Brunton et al., 2013 [14]	In vivo	P_11_-4 is safe and associated with significant enamel regeneration by promoting mineral deposition within the subsurface tissue
Kind et al., 2017 [22]	In vitro	P_11_-4 diffuses into the depth of carious lesion
Metwally et al., 2017 [42]	In vitro/in vivo	Self-assembling peptides (Curodont Repair) were successful as a remineralizing agent in young permanent teeth with white spot lesions
Schlee et al., 2018 [30]	Practice-based case series	Radiographic and digital subtraction analyses suggested that initial proximal carious lesions can regress after treatment with P_11_-4
Alkilzy et al., 2018 [23]	Randomized clinical trial	P_11_-4 in combination with fluoride application is a simple, safe, and effective noninvasive treatment for early carious lesions, which is superior to the fluoride alone
Sindhura et al., 2018 [16]	In vitro	SAP P_11_-4 exhibited superior remineralization with uniform mineral deposition compared to CPP/ACP at the 3 month interval
Jablonski-Momeni et al., 2019 [43]	In situ	The self-assembling peptide P_11_-4 may represent a preventive or therapeutic measure in accompanying orthodontic treatments to prevent the formation of white spots around orthodontic brackets or to treat and mask them
Doberdoli et al., 2020 [31]	Randomized clinical trial	SAP P_11_-4, applied in combination with fluoride varnish or twice-weekly SAPM, was a superior treatment for early caries compared to fluoride varnish alone
Bröseler et al., 2020 [26]	Randomized clinical trial	The size of early carious lesions treated with P_11_-4 was significantly reduced; this result was superior to that of fluoride varnish
Sedlakova et al., 2020 [33]	Controlled, blinded split-mouth clinical trial	P_11_-4 supports the formation of de novo hydroxyapatite crystals deep within and throughout the carious lesion body; it offers the clinician a new, effective, non-aerosol-generating, and noninvasive treatment option
Welk et al., 2020 [34]	Controlled clinical trial	Treatment of white spot lesions with self-assembling peptide P_11_-4 led to superior remineralization of the subsurface lesions compared with the control teeth
Kobeissi et al., 2020 [36]	Split-mouth controlled trial	P_11_-4 showed superiority in treatment of post-orthodontic lesions due to its guided enamel regeneration potential
Aparna et al., 2022 [44]	Systematic review and meta-analysis	In vitro and in vivo studies showed evidence of superior biomimetic remineralization in the P_11_-4 group compared to other remineralizing agents
Gohar et al., 2023 [35]	Randomized clinical trial	Biomimetic remineralization promoted by self-assembling peptide P_11_-4 achieved successful subsurface remineralization, making the material a promising guide to lesion regression in post-orthodontic therapy

**Table 2:** Indications and limitations for minimally invasive caries treatment with self-assembling peptide P_11_-4.

Indications and Conditions for Treatment with P_11_-4	Contraindications and Limitations for Treatment with P_11_-4
Early carious lesion without cavitation	Carious lesion with cavitation
Patients with no/low compliance with tooth brushing and dental hygiene	Good patient compliance with dental hygiene
Patients with moderate caries risk and activity	Patients with low caries risk
Age groups accompanied with caries activity (adolescents and young adults)	Elderly patients with slow caries progression or already arrested lesions
Lesion progression in spite of preventive measures	Allergy to the product

## Data Availability

Not applicable.
